# Accidental intravitreal injection of a veterinary inactivated FMD vaccine resulting in noninfectious intraocular inflammation: a case report

**DOI:** 10.3389/fmed.2026.1796518

**Published:** 2026-05-22

**Authors:** Xin Zhang, Peipei Jia, Zheng Fan, Xiaolu Cao, Zhimin Chen

**Affiliations:** Hebei Key Laboratory of Ophthalmology, Hebei Eye Hospital, Hebei Ophthalmology Research Institute, Xingtai, China

**Keywords:** FMD vaccine, intraocular liquid foreign body, intravitreal injection, noninfectious intraocular inflammation, uveitis

## Abstract

Penetrating ocular trauma is the main cause of irreversible loss of unilateral vision in young men. Among its rare presentations, accidental intravitreal injection of non-human biological agents poses unique diagnostic and therapeutic challenges. The bivalent inactivated vaccine against swine foot-and-mouth disease (FMD) serotype O and A is currently an effective means of preventing swine FMD virus infection and is strictly prohibited for human use. To date, no clinical reports have been observed regarding intraocular exposure (particularly injection into the vitreous cavity) of this vaccine. This article presents a case of severe noninfectious intraocular inflammation resulting from accidental injection of the bivalent inactivated vaccine against swine FMD serotype O and A into the vitreous cavity, aiming to analyze its unique pathogenesis and summarize diagnostic and therapeutic lessons.

## 1 Introduction

Penetrating ocular trauma refers to the complete penetration of the eyeball wall by an external force (such as a sharp instrument, etc.) to form a full layer of wounds, making the intraocular structure communicate with the outside world, which is the main cause of irreversible unilateral visual impairment in young men (1). The bivalent inactivated FMD vaccine against serotypes O and A is widely applied for the prevention of FMD virus infection in swine (2). This vaccine mainly consists of a mineral oil-based adjuvant, inactivated viral antigens, and chemical inactivating agents, and functions by eliciting both humoral and cellular immune responses in pigs to interrupt viral transmission. If this vaccine is accidentally injected into the human body, the components in the vaccine will trigger an immune inflammatory response in the organism (3, 4). To date, no clinical reports have been observed regarding intraocular exposure (particularly injection into the vitreous cavity) of this vaccine. Here, we report a case in which inadvertent intravitreal administration of a porcine bivalent inactivated FMD vaccine led to severe noninfectious intraocular inflammation. This report aims to analyze the underlying pathogenic mechanisms and to summarize key points from the diagnostic and therapeutic course.

## 2 Case report

A 48-year-old man was admitted with a 48-day history of redness in the left eye and a 20-day history of blurred vision. The patient reported that 48 days prior to admission, the skin of the lower eyelid had been accidentally punctured by an automatic injection needle preloaded with a porcine bivalent inactivated FMD vaccine (serotype O and A; OHM/O2 strain + AKT-III strain). Eyelid and conjunctival ecchymosis with edema developed shortly thereafter. He was evaluated at a local hospital and received antibiotic therapy. The specific antibiotics administered at the local hospital prior to admission could not be identified due to the lack of accessible medical records. Twenty days later, blurred vision appeared, for which he self-administered oral anti-inflammatory medications. As symptoms progressively worsened, he presented to our institution. The patient had no significant past medical history.

**FIGURE 1:**
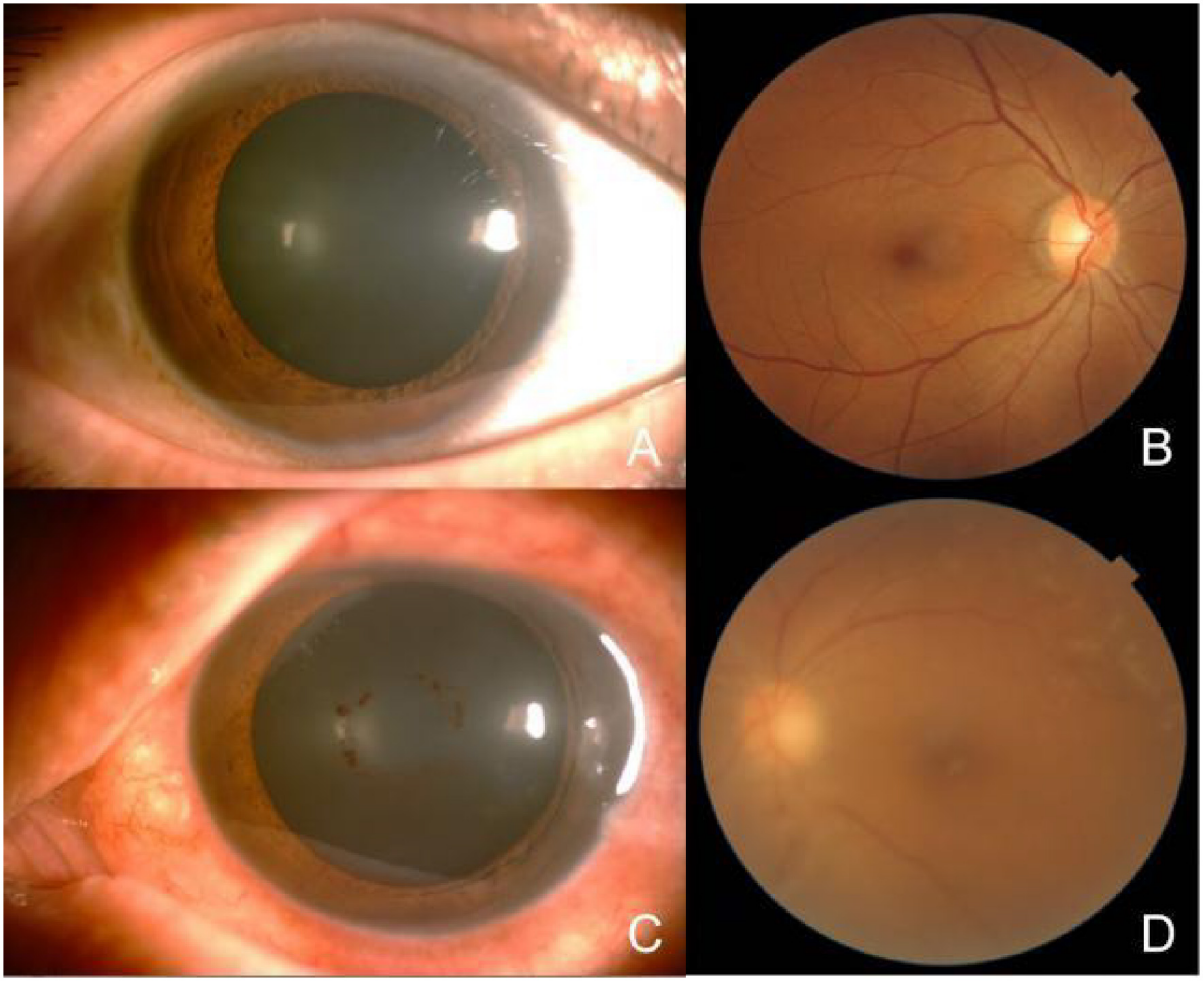
Photography of the anterior segment and fundus of both eyes. **(A,B)** The anterior and posterior segments of the right eye were within normal limits. **(C,D)** In the left eye, conjunctival congestion and edema, with significant edema in the inferior conjunctiva. Mild corneal edema was present. The anterior chamber contained inflammatory cells (++), with a marked flare (+++). The lens cortex showed irregular opacification. Dense vitreous haze was observed, with abundant inflammatory cells (+++), and the retina could be faintly visualized *in situ*.

On admission, general physical examination was unremarkable, and no abnormalities were detected on cardiopulmonary assessment. Ophthalmic examination revealed best-corrected visual acuity of 0.8 in the right eye and 0.04 in the left eye. Non-contact tonometry showed intraocular pressure of 18 mmHg in the right eye and 60 mmHg in the left eye. The anterior and posterior segments of the right eye were within normal limits. In the left eye, marked edema of the lower eyelid skin was noted, accompanied by conjunctival hyperemia and chemosis, with pronounced inferior conjunctival edema. Mild corneal edema was present. The anterior chamber contained inflammatory cells (++), with a marked flare (+++). The iris exhibited 360° posterior synechiae, and a small amount of exudation was visible at the pupillary margin. The lens cortex showed irregular opacification. Dense vitreous haze was observed, with abundant inflammatory cells (+++), and the retina could be faintly visualized *in situ* (Figure 1).

**FIGURE 2:**
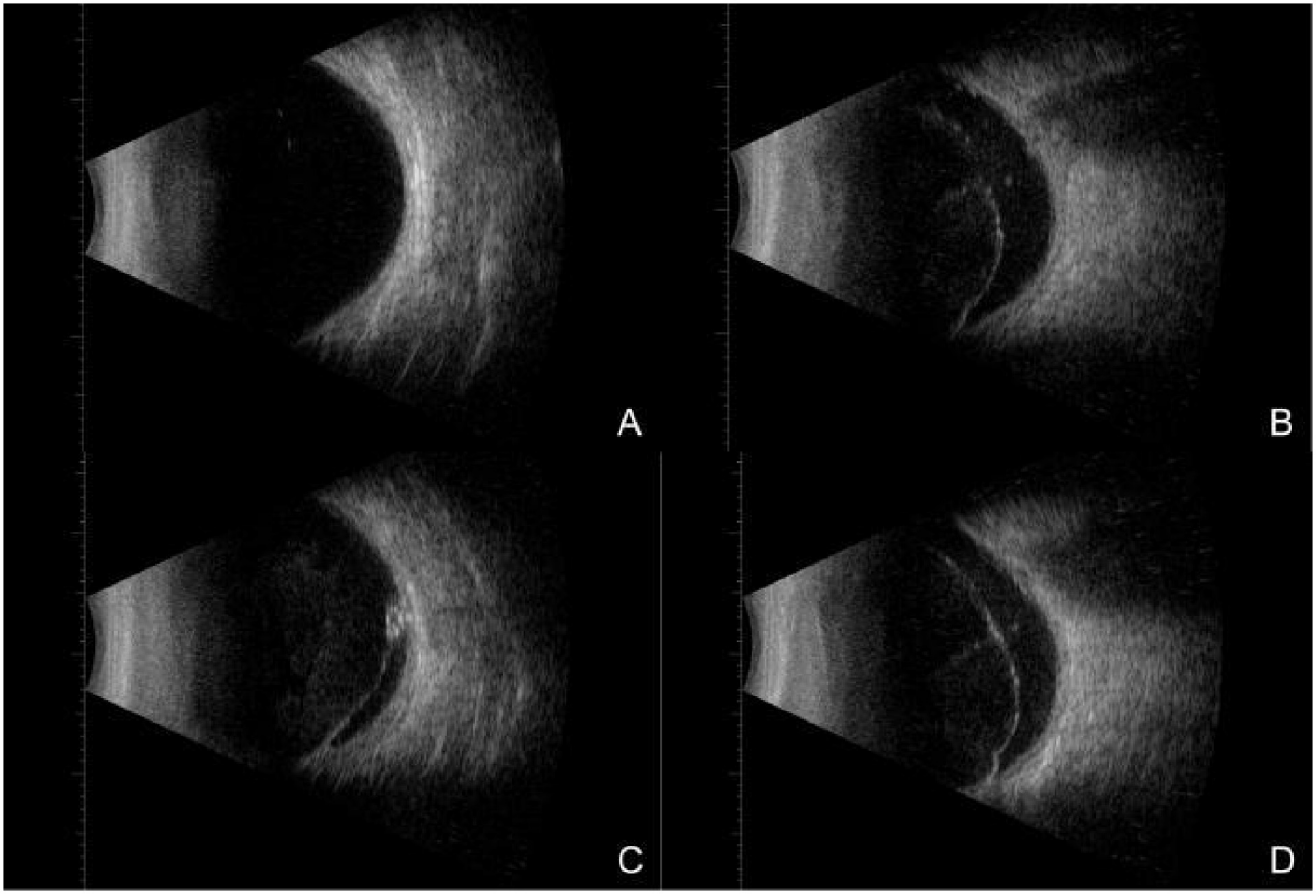
On bilateral B-scan ultrasonography at initial presentation, **(A)** demonstrated punctate low-level echoes within the vitreous cavity of the right eye, with no evident retinal abnormalities. **(B–D)** Revealed relatively dense punctate and flocculent low-level echoes and continuous membranous structures within the vitreous cavity of the left eye, with adhesions to the nasal peripheral ocular wall and patchy strong echoes detected adjacent to the globe wall; no definitive signs of retinal detachment were observed.

**FIGURE 3:**
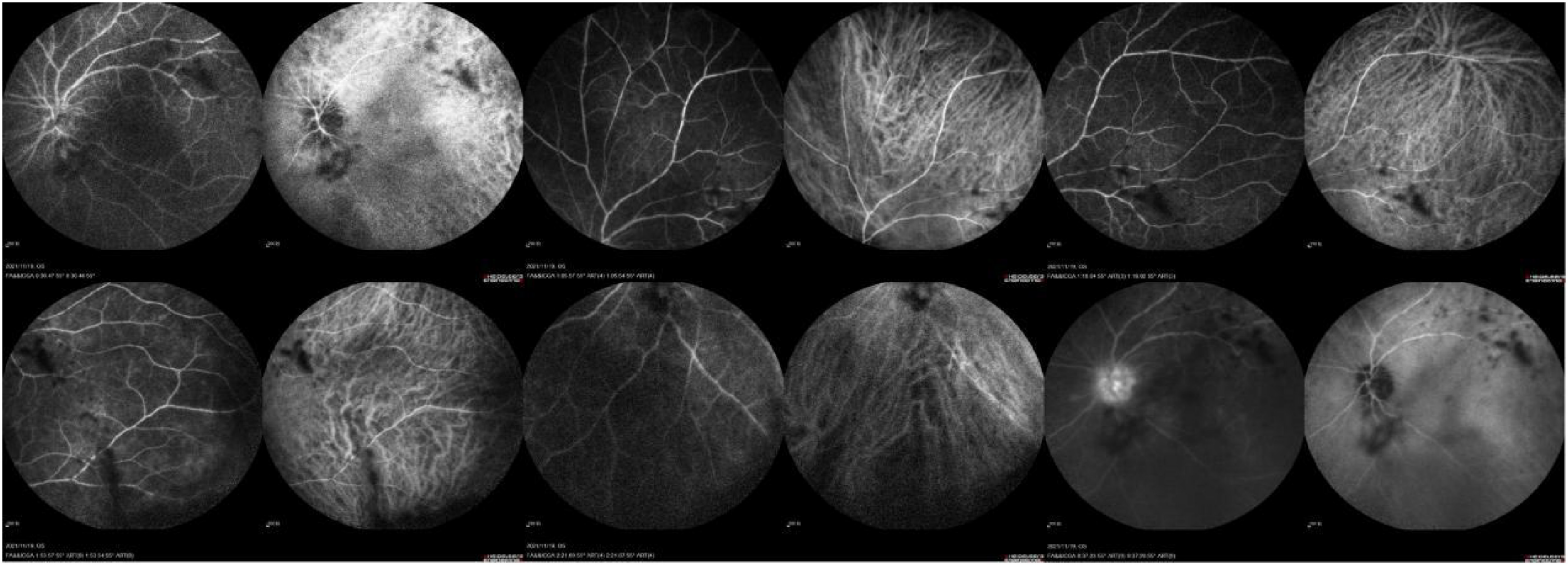
FFA and ICGA of the left eye revealed poor clarity of the refractive media, tortuous and dilated retinal veins with irregular caliber and segmental staining, diffuse leakage from retinal capillaries, indistinct optic disc margins with intense late-phase hyperfluorescence, reduced background fluorescence in the inferior peripheral retina, and obscured retinal architecture.

Routine laboratory investigations, including complete blood count, urinalysis, C-reactive protein, liver and renal function tests, and electrolyte analysis, showed no significant abnormalities. B-scan ultrasonography demonstrated vitreous opacities in the right eye, while the left eye exhibited vitreous opacification with organized membranous echoes, posterior vitreous detachment, and strong intraocular echoes, raising the suspicion of an intraocular foreign body in conjunction with clinical findings (Figure 2). Fundus fluorescein angiography (FFA) and indocyanine green angiography (ICGA) of the left eye revealed poor clarity of the refractive media, tortuous and dilated retinal veins with irregular caliber and segmental staining, diffuse leakage from retinal capillaries, indistinct optic disc margins with intense late-phase hyperfluorescence, reduced background fluorescence in the inferior peripheral retina, and obscured retinal architecture (Figure 3). Based on these findings, the patient was diagnosed with a penetrating eye injury of left eye, intraocular liquid foreign body in the left eye, uveitis, secondary glaucoma, and complicated cataract.

**FIGURE 4:**
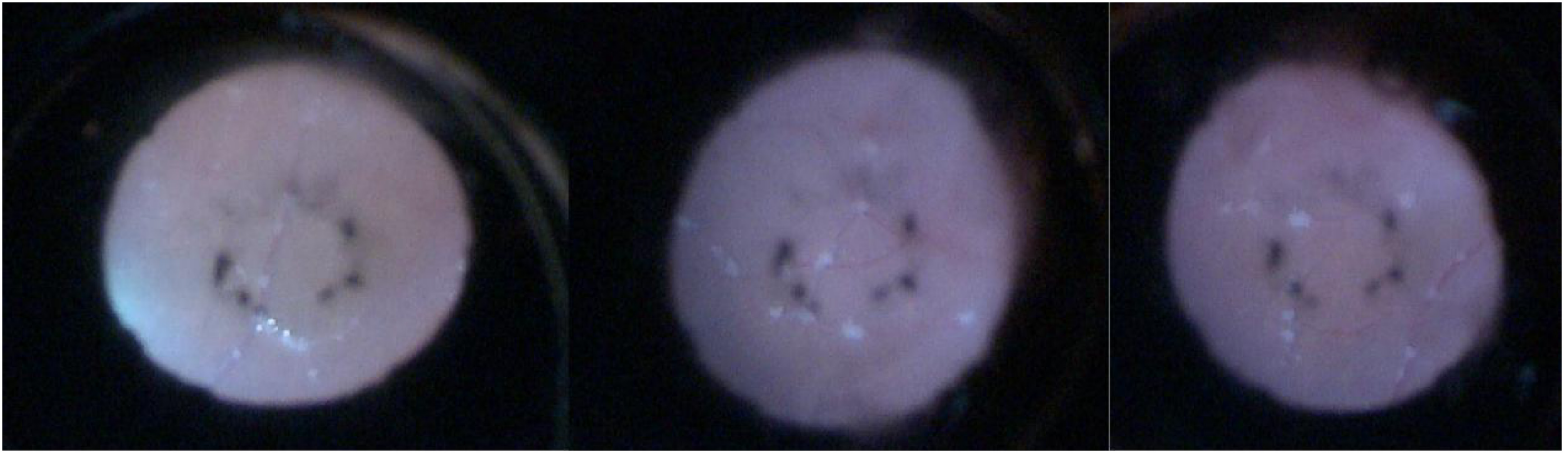
Intraoperatively, milky white droplets consistent with the injected agent were identified within the vitreous cavity, the inferotemporal peripheral retina, and the superior retina.

Topical antibiotic and intraocular pressure-lowering therapies were initiated after admission, but no meaningful clinical improvement was observed. The patient subsequently underwent pars plana vitrectomy combined with removal of the intraocular liquid foreign material and silicone oil tamponade under general anesthesia. Intraoperatively, the vitreous cavity appeared turbid, with a pale optic disc and indistinct margins. Milky white droplets consistent with the injected agent were identified within the vitreous cavity, the inferotemporal peripheral retina, and the superior retina (Figure 4). Scattered retinal hemorrhages were present, with a sinus tract noted in the inferotemporal retina and peripheral retinal detachment. Vitrectomy was performed to remove the intraocular liquid foreign body, followed by endolaser photocoagulation to the affected retinal areas. A total of 5.5 mL of silicone oil was injected, achieving retinal reattachment.

**FIGURE 5:**
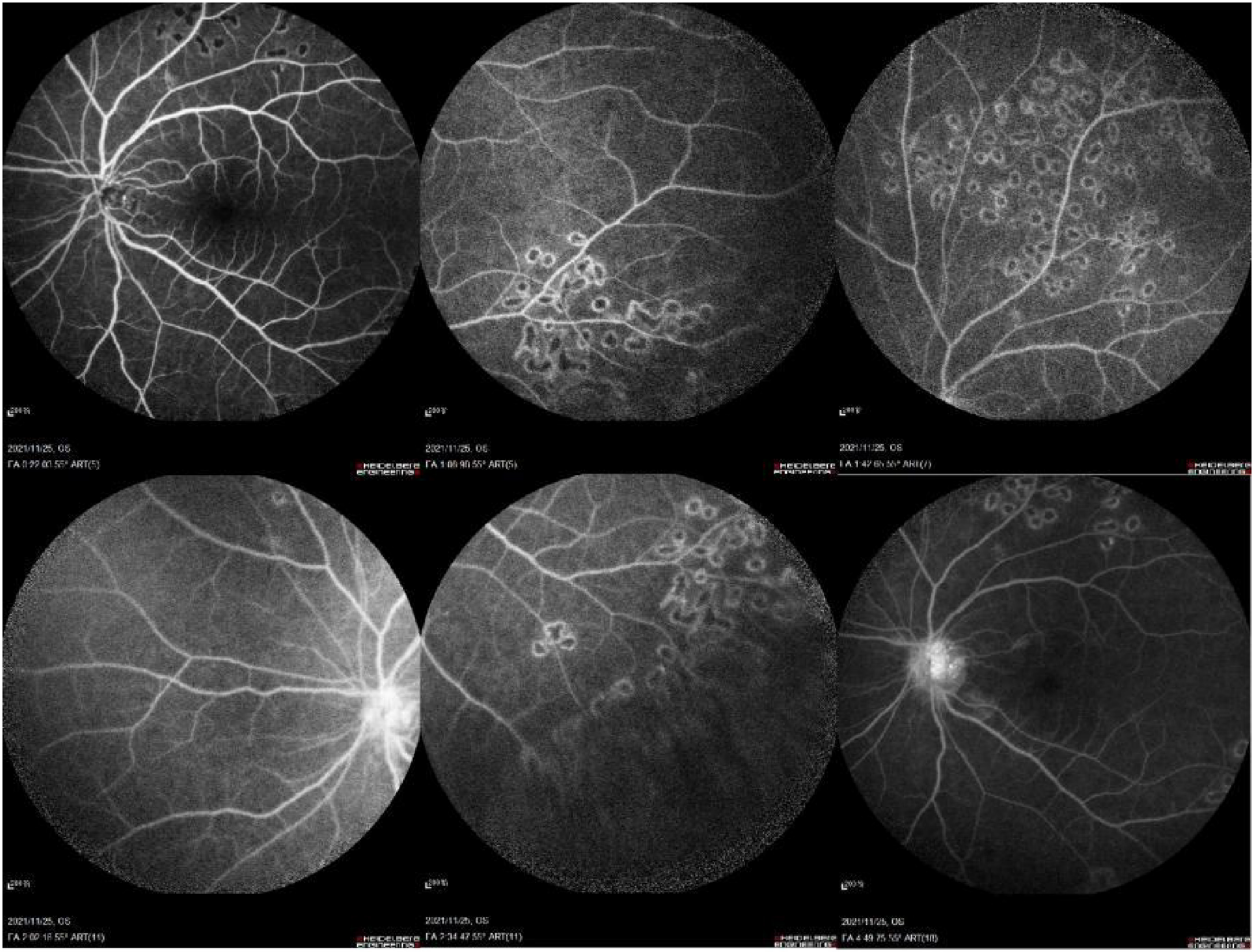
First postoperative FFA of the left eye: left eye silicone oil eye, left eye retinal photocoagulation postoperative; left eye retinal venous tortuosity, capillary dilation and leakage at the optic disc, and patchy atrophic scars corresponding to laser photocoagulation in the superotemporal and inferotemporal peripheral retina.

Postoperatively, topical antibiotics were administered for infection prophylaxis, along with mydriatic therapy. Pre-discharge FFA demonstrated persistent retinal venous tortuosity, capillary dilation and leakage at the optic disc, and patchy atrophic scars corresponding to laser photocoagulation in the superotemporal and inferotemporal peripheral retina (Figure 5). Visual acuity in the left eye improved to 0.12, with a corrected visual acuity of 0.3 using +5.00 diopters sphere. The patient was discharged in stable condition.

**FIGURE 6:**
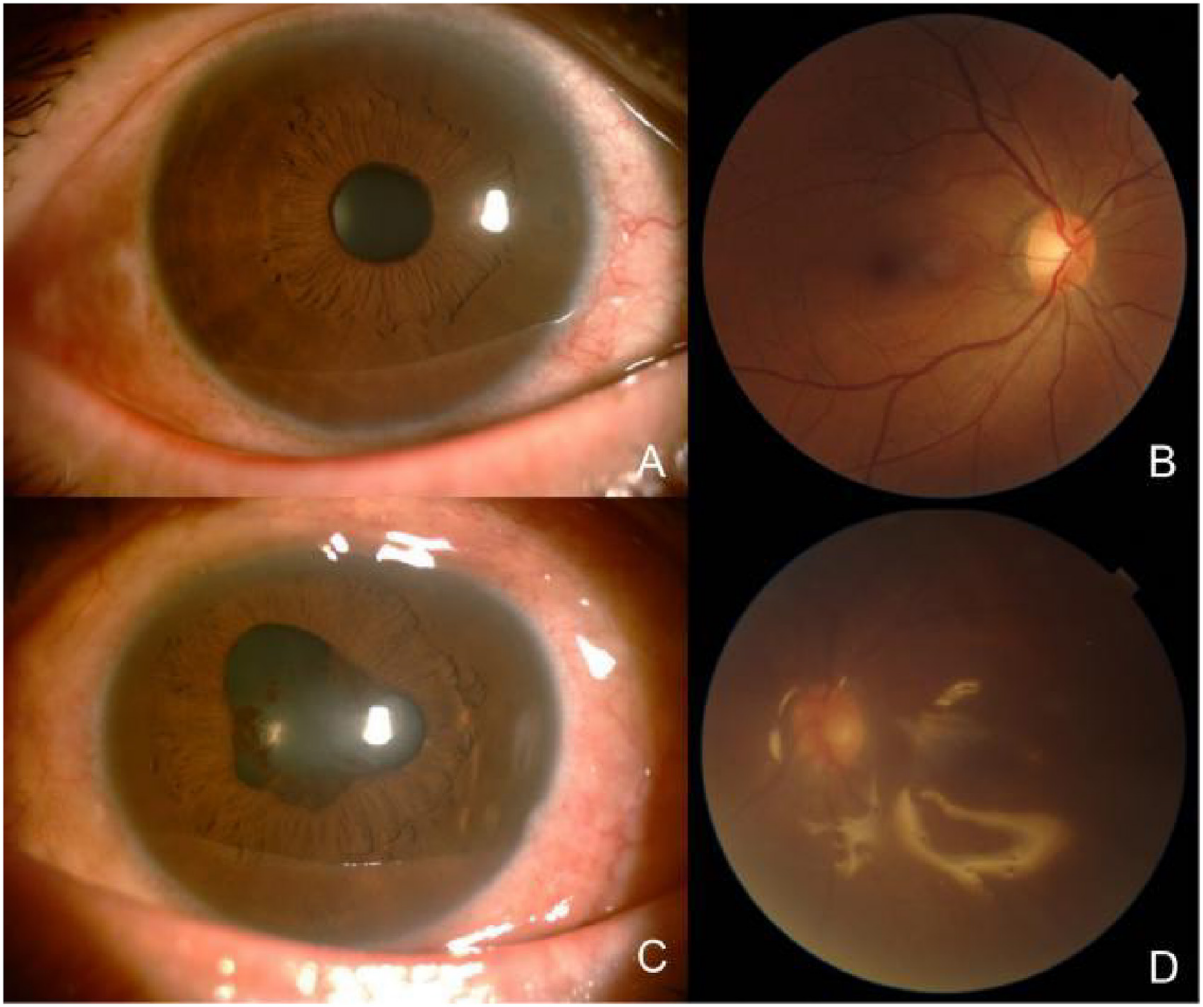
Second hospitalization with anterior segment and fundus photography of both eyes. **(A,B)** No abnormalities were detected in the right eye. **(C,D)** In the left eye, marked mixed conjunctival hyperemia (+++) was present, with gray-white dust-like keratic precipitates (+). The anterior chamber depth was within normal limits, with mild floating inflammatory material (+) and anterior chamber flare (+). The pupil was irregular, with a horizontal diameter of approximately 4 mm, and irregular posterior synechiae of the iris were observed. Opacification of the lens cortex and posterior capsule was evident. Silicone oil filling was visible within the vitreous cavity. The optic disc appeared normal in color with clear margins. Retinal veins were dilated and tortuous. The macular architecture was indistinct, and the foveal reflex was absent.

**FIGURE 7:**
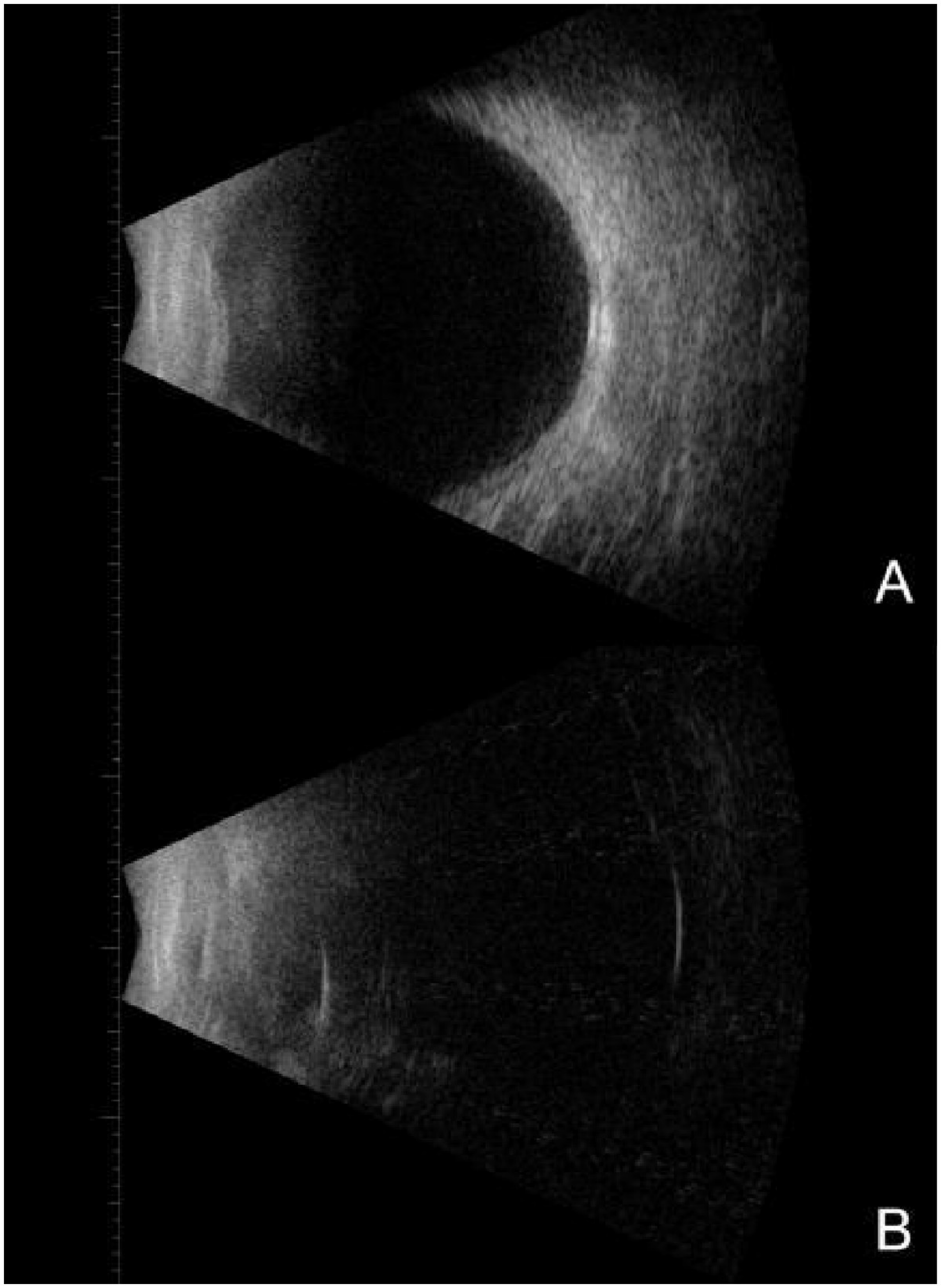
During the second hospitalization, bilateral B-scan ultrasonography showed punctate low-level vitreous echoes in the right eye without apparent retinal pathology **(A)**. In the left eye, the posterior interface of silicone oil was identified in the supine position, while a small amount of true ocular wall echo was detected in the lateral position. Within the observable range, no clear evidence of retinal detachment was noted **(B)**.

**FIGURE 8:**
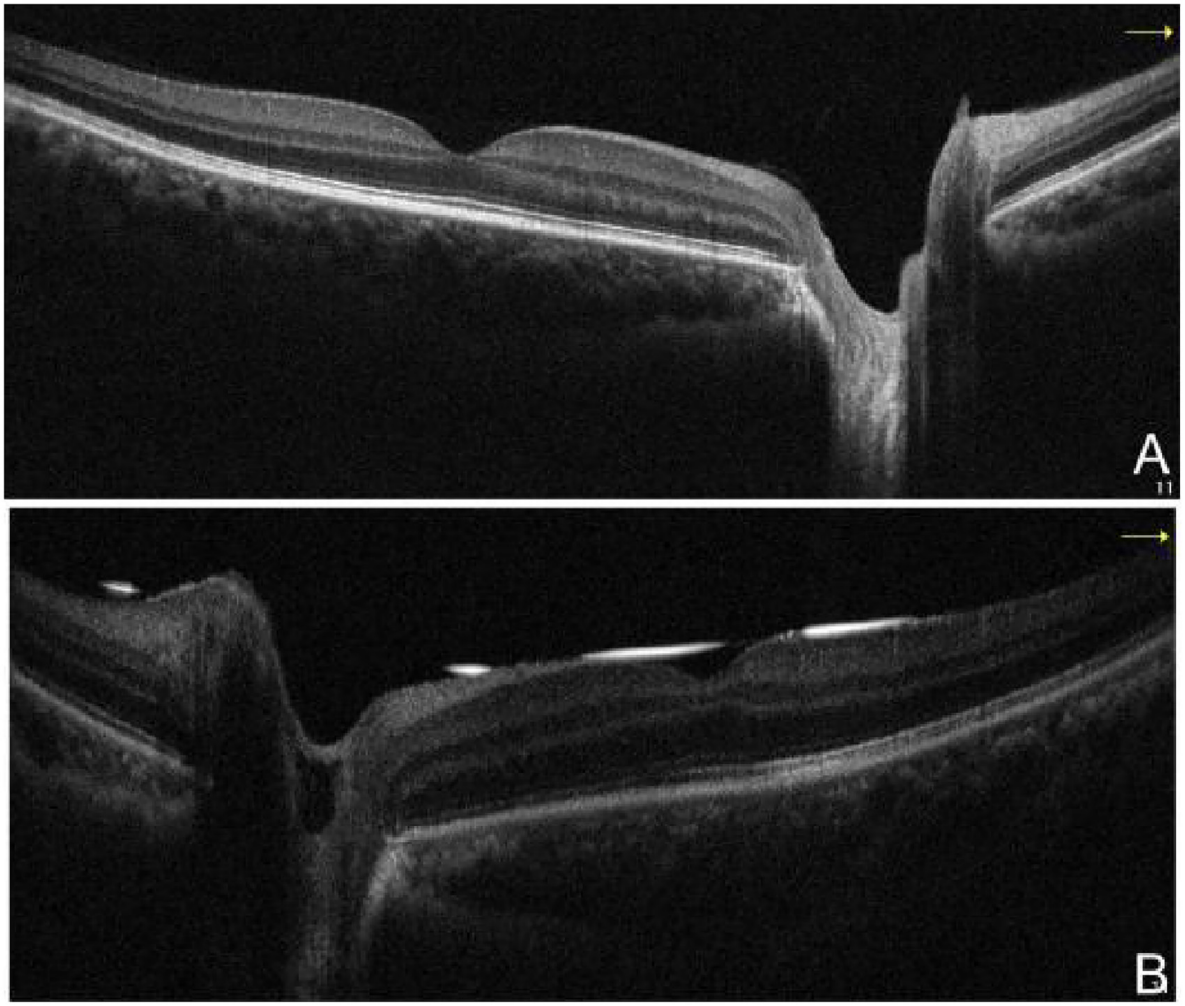
Second hospitalization with bilateral optical coherence tomography. **(A)** right eye, **(B)** left eye. That demonstrated largely preserved macular structural integrity in both eyes.

Two months after discharge, the patient was readmitted because of the sudden onset of redness, ocular pain, and blurred vision in the left eye, accompanied by excessive tearing. Ophthalmic examination showed visual acuity of 1.0 in the right eye and 0.06 in the left eye. Best-corrected visual acuity was 1.0 in the right eye with plano correction and 0.8 in the left eye with +7.00 diopters sphere. Non-contact tonometry revealed intraocular pressure of 18 mmHg in the right eye and 13 mmHg in the left eye. No abnormalities were detected in the right eye. In the left eye, marked mixed conjunctival hyperemia (+++) was present, with gray-white dust-like keratic precipitates (+). The anterior chamber depth was within normal limits, with mild floating inflammatory material (+) and anterior chamber flare (+). The pupil was irregular, with a horizontal diameter of approximately 4 mm, and irregular posterior synechiae of the iris were observed. Opacification of the lens cortex and posterior capsule was evident. Silicone oil filling was visible within the vitreous cavity. The optic disc appeared normal in color with clear margins. Retinal veins were dilated and tortuous. The macular architecture was indistinct, and the foveal reflex was absent (Figure 6). Ancillary examinations showed vitreous opacities in the right eye and postoperative silicone oil-related echoes in the left eye on B-scan ultrasonography (Figure 7). Optical coherence tomography demonstrated largely preserved macular structural integrity in both eyes (Figure 8). Residual intraocular liquid foreign material from the prior episode was suspected, with persistent porcine vaccine components within the eye considered responsible for a secondary immune-mediated inflammatory response. Topical antibiotics and mydriatic agents were administered to alleviate inflammation, and oral prednisone was prescribed for systemic anti-inflammatory therapy. Following treatment, the patient reported resolution of redness and pain in the left eye, improved visual clarity, and a reduction in intraocular inflammatory signs. He was discharged with instructions to taper oral prednisone gradually to maintain suppression of the inflammatory response.

**FIGURE 9:**
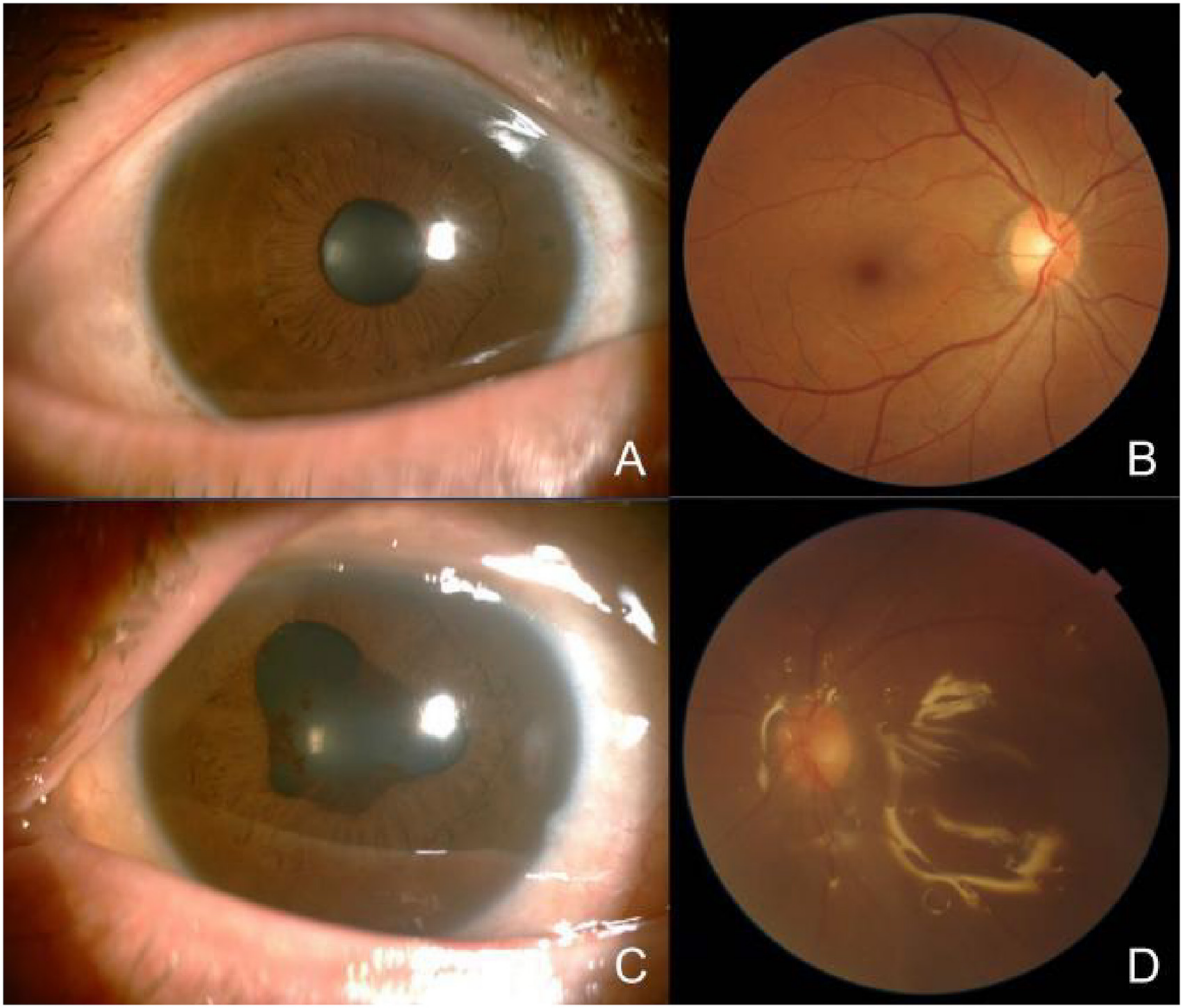
Third hospitalization with anterior segment and fundus photography of both eyes. **(A,B)** Examination revealed no abnormalities in the anterior or posterior segments of the right eye. **(C,D)** In the left eye, irregular posterior synechiae of the iris persisted, with opacification of the lens cortex and posterior capsule. Silicone oil remained present in the vitreous cavity. Fundus examination showed a normally colored optic disc with well-defined margins, mildly tortuous retinal veins, and absence of the foveal light reflex.

**FIGURE 10:**
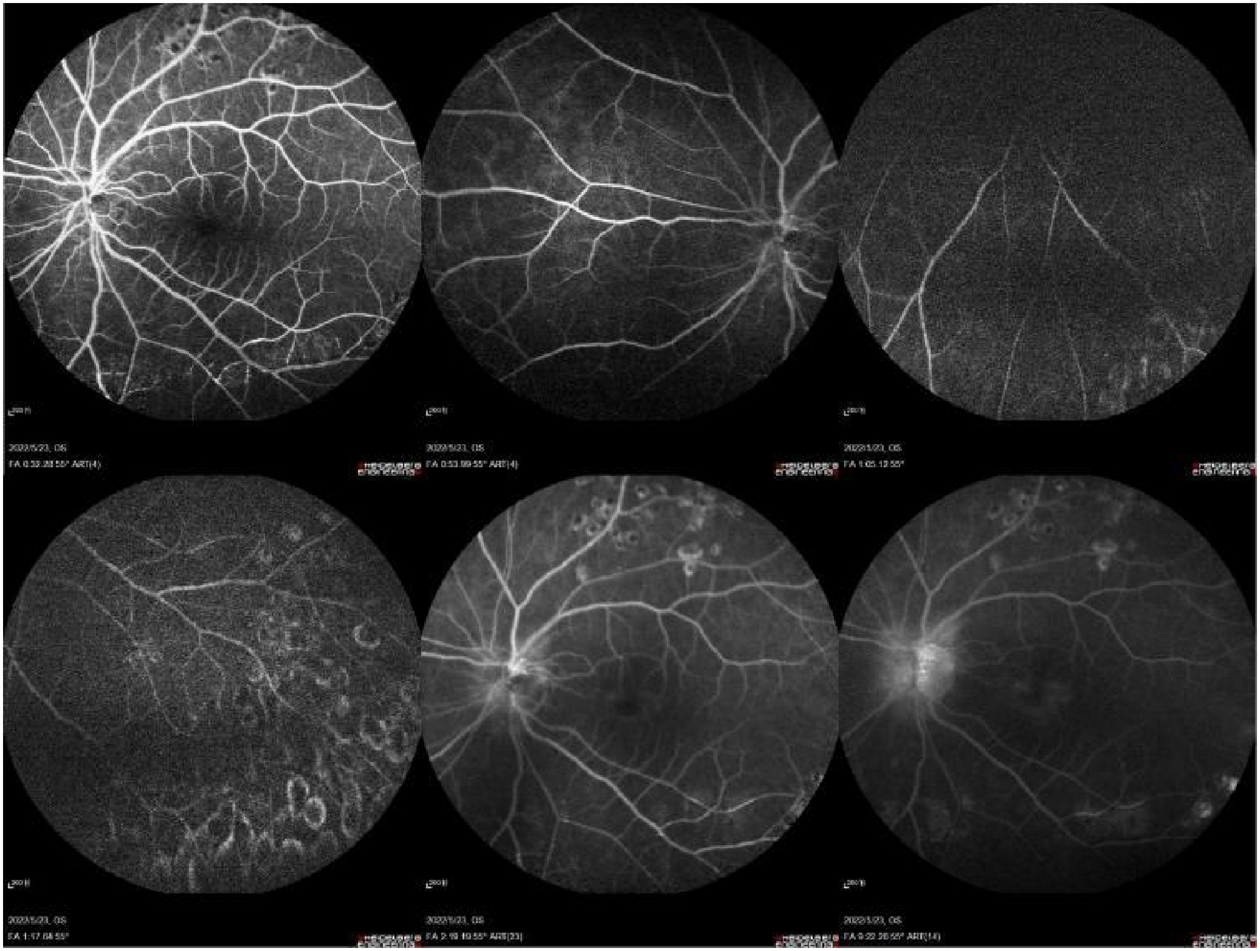
Third hospitalization for left eye FFA performed: despite suboptimal clarity of the refractive media, demonstrated indistinct optic disc margins, capillary dilation with leakage, tortuous and dilated retinal veins with irregular caliber and segmental vessel wall staining, scattered patchy atrophic scars corresponding to prior laser photocoagulation in the peripheral retina, capillary leakage in the macular region, and intense late-phase hyperfluorescence of the optic disc.

Three months later, silicone oil removal was planned. Examination revealed no abnormalities in the anterior or posterior segments of the right eye. In the left eye, irregular posterior synechiae of the iris persisted, with opacification of the lens cortex and posterior capsule. Silicone oil remained present in the vitreous cavity. Fundus examination showed a normally colored optic disc with well-defined margins, mildly tortuous retinal veins, and absence of the foveal light reflex (Figure 9). FFA of the left eye, performed despite suboptimal clarity of the refractive media, demonstrated indistinct optic disc margins, capillary dilation with leakage, tortuous and dilated retinal veins with irregular caliber and segmental vessel wall staining, scattered patchy atrophic scars corresponding to prior laser photocoagulation in the peripheral retina, capillary leakage in the macular region, and intense late-phase hyperfluorescence of the optic disc (Figure 10).

**FIGURE 11:**
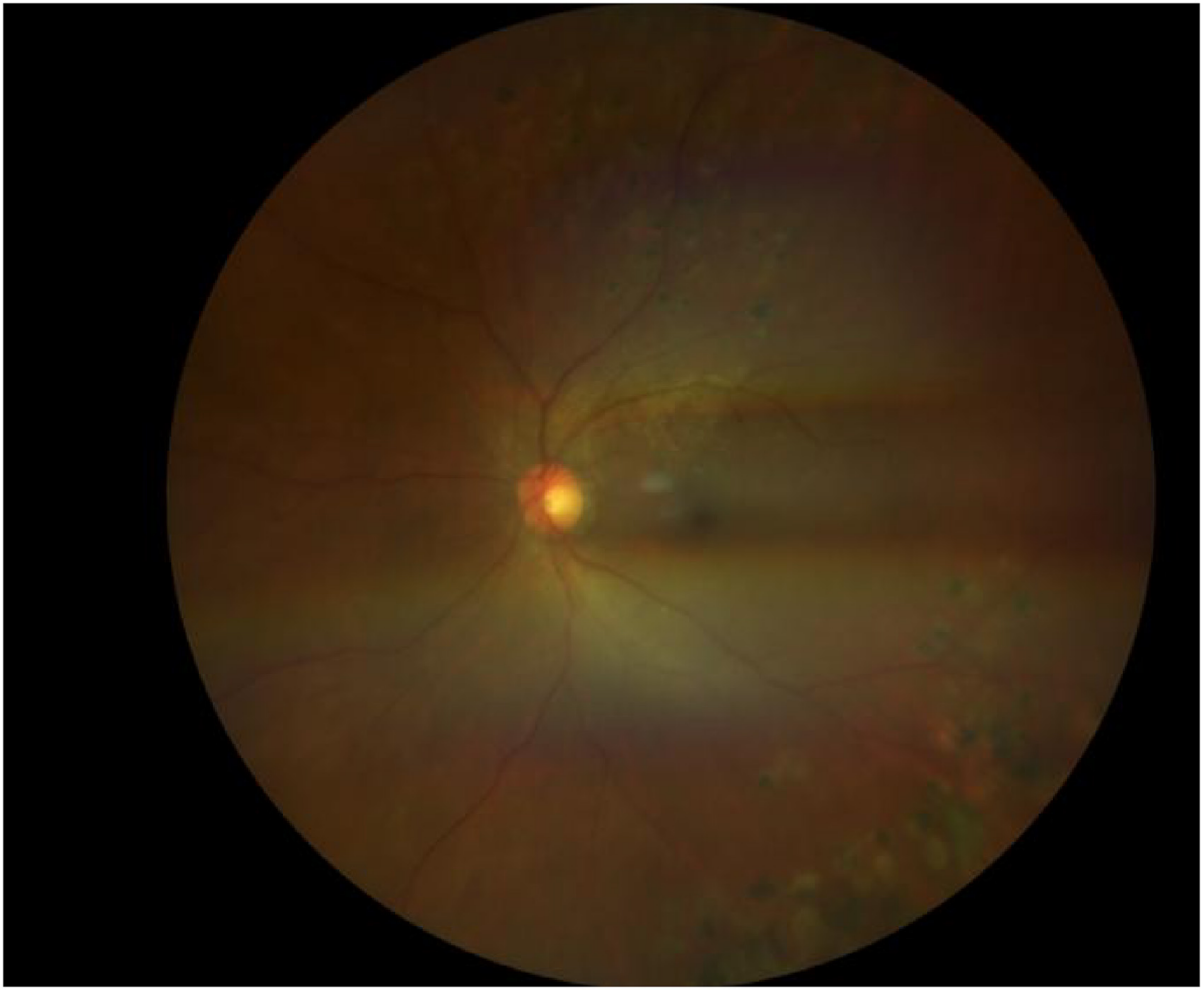
Fundus after silicone oil extraction in the left eye showed: a clear optic disc margin with a pale pink coloration, largely normal retinal vascular configuration and proportions, and residual old laser photocoagulation scars. The foveal reflex remained absent.

Under general anesthesia, the patient underwent phacoemulsification with intraocular lens implantation combined with silicone oil removal in the left eye. Intraoperatively, the optic disc margins were clear but pale in color. Extensive proliferative membranes with circumferential contraction were observed in the peripheral retina, and proliferative vitreoretinopathy was present inferiorly. Small residual white droplets consistent with retained vaccine material were still visible in the peripheral retina. Residual emulsified silicone oil was removed as thoroughly as possible. Incisions were made in the inferior and inferonasal peripheral proliferative retina, additional retinal photocoagulation was applied, and silicone oil tamponade was reintroduced. Postoperative recovery was uneventful, and the clinical condition remained stable. Eighteen months later, silicone oil removal was performed again in the left eye. Postoperatively, fundus examination showed a clear optic disc margin with a pale pink coloration, largely normal retinal vascular configuration and proportions, and residual old laser photocoagulation scars. The foveal reflex remained absent (Figure 11). Intraocular pressure remained mildly elevated after surgery. Although gonioscopy confirmed an open anterior chamber angle, topical antiglaucoma medications were continuously administered in the left eye to maintain intraocular pressure control. Visual recovery was favorable, with uncorrected visual acuity improving to 0.6. The progression timeline of the patient’s disease is shown in Table 1.

**TABLE 1:** Summary of clinical events.

Time point	Event	Key findings
Day 0	Accidental needlestick	Lower eyelid punctured by FMD vaccine needle; eyelid ecchymosis/edema.
Day 0–20	Local treatment	Antibiotics (unknown); day 20: blurred vision.
Day 48	First admission	BCVA 0.04, IOP 60 mmHg, anterior chamber cells/flare, 360° posterior synechiae, vitreous haze; diagnosed: penetrating injury, intraocular liquid foreign body, uveitis, secondary glaucoma, cataract.
Surgery	Vitrectomy + foreign material removal + silicone oil	Intraop: milky white droplets in vitreous/retina, retinal hemorrhages, sinus tract, detachment; endolaser. BCVA improved to 0.12 (0.3 with +5.00 D); FFA: leakage, laser scars; discharged stable.
2 months post-discharge	Readmission (recurrence)	Redness, pain, blurred vision; BCVA 0.06 (0.8 with +7.00 D), anterior chamber cells/flare, synechiae, silicone oil in situ; suspected residual vaccine-induced inflammation. Topical antibiotics, mydriatics, oral prednisone → resolved; discharged on tapering prednisone.
3 months later	Phaco+IOL+silicone oil removal	Synechiae, lens opacity, silicone oil in situ, normal disc, absent foveal reflex. Intraop: pale disc, proliferative membranes, PVR, residual white droplets; silicone oil reintroduced.
18 months later	Second silicone oil removal	Post-op: clear disc margin (pale pink), normal vessels, old laser scars, absent foveal reflex; IOP mildly elevated; UCVA 0.6.

## 3 Discussion

Ocular trauma is a global public health problem and a common cause of monocular blindness in young men. Penetrating ocular trauma refers to ocular injury in which the eyeball enters the wound but there is no exit wound. The common causes are blunt wounds, sharp wounds and intraocular foreign bodies (1, 5). Among its rare presentations, accidental intravitreal injection of non-human biological agents poses unique diagnostic and therapeutic challenges. FMD is an acute, highly virulent, and highly contagious infectious disorder caused by the FMD virus, predominantly affecting cloven-hoofed animals such as pigs, cattle, and sheep (6). At present, vaccination with inactivated [The main components are mineral oil adjuvant and inactivated viral protein (7)] FMD vaccines remains the most practical and effective strategy for disease prevention in swine, cattle, and sheep. Such components may directly damage intraocular tissues through mechanical injury and chemical toxicity, while foreign material-induced immune and inflammatory responses further contribute to visual impairment.

Mineral oil adjuvants, primarily composed of white mineral oil, are petrochemical products that cannot be metabolized in the eye. They persist as long-standing foreign materials, causing continuous mechanical irritation, interference with intraocular metabolic processes, and potential obstruction of the anterior chamber angle, which may lead to secondary glaucoma. Although research on the ocular toxicity of mineral oil is limited, studies have found that silicone oil—a similar compound—can suppress proliferation of human trabecular meshwork cells and trigger ferroptosis and fibrotic responses (8). As a veterinary formulation, mineral oil adjuvants are expected to exert substantially greater toxicity than medical-grade silicone oil. These effects may be associated with the elevated intraocular pressure and secondary glaucoma observed in the patient’s left eye.

In the present case, the inflammatory response did not represent a simple sterile reaction, but rather an immune-mediated process likely triggered by vaccine components (9). Adjuvants are designed to stimulate and amplify immune responses in animals (10); when introduced into the human eye, the resulting immune activation is expected to be even more intense. Therefore, the mechanism may involve the activation of intraocular immune pathways by mineral oil adjuvants, the disruption of the immune-privileged status of ocular tissues (e.g., the vitreous cavity and retina), and the promotion of a localized pro-inflammatory microenvironment. This process facilitates infiltration by neutrophils and macrophages (11), enhances antigen presentation of inactivated viral components by intraocular antigen-presenting cells, and stimulates both humoral and cellular immune responses. Concurrently, antigen–antibody complexes may form within the eye (9), culminating in severe intraocular inflammation manifested by conjunctival and corneal edema, iris synechiae, and marked accumulation of inflammatory cells in the anterior chamber and vitreous cavity.

Mineral oil adjuvant is a common component in various veterinary vaccines, and multiple previous studies have documented its ability to induce immune responses in humans. Literature review identified several documented cases of accidental human inoculation with FMD vaccine or analogous oil-adjuvanted veterinary vaccines (Table 2). Jones reported three cases of Footvax (oil-adjuvanted FMD vaccine) self-inoculation: one thigh injection caused extensive chronic granulomatous inflammation requiring repeated surgical debridements, and two hand injections led to sterile abscesses requiring drainage and corticosteroids (12). O’Neill described a bovine vaccine injection into a finger that, despite repeated debridement, resulted in tissue ischemia and amputation (3). A systematic review of 59 needlestick injuries with veterinary biologics found that 34% involved oil-adjuvant vaccines, the hand was the most common site (67%), and 14 patients had residual sequelae (13). A prospective study confirmed that mineral oil adjuvant is an independent risk factor for both early and late local complications (14).

**TABLE 2:** Reported cases of accidental FMD vaccine inoculation in humans, categorized by injection location and clinical manifestations.

Injection location	Vaccine type/adjuvant	Clinical manifestations	Outcome/Sequelae	Reference(s)
Thigh (intramuscular)	Footvax (FMD vaccine, oil-adjuvanted)	Extensive chronic granulomatous inflammation	Required major surgical debridements	Jones (12)
Hand (subcutaneous)	Footvax (FMD vaccine, oil-adjuvanted)—two cases	Chronic inflammation, sterile abscess formation	Required surgical drainage and corticosteroids	Jones (12)
Little finger (subcutaneous)	Bovine vaccine (oil-adjuvanted, analogous formulation)	Increased compartment pressure, signs of ischemia	Amputation of digit despite repeated surgical debridement	O’Neill et al. (3)
Various (hand: 67%, leg: 28%, other: 5%)	Multiple veterinary vaccines, including oil-adjuvanted types	Granulomatous inflammation, sterile abscess, tissue ischemia	14 of 30 patients with residual sequelae; 1 death; hospitalization (median 3 days); surgical intervention in 25/59 cases	Buswell et al. (14)
Various	Veterinary vaccines with mineral oil adjuvant	Mineral oil adjuvant identified as independent risk factor for early and late locoregional complications	Absence from work significantly correlated with injection site and presence of mineral oil adjuvant	Meyer et al. (15)

These reports consistently demonstrate that mineral oil adjuvant acts as a persistent foreign body, eliciting chronic granulomatous inflammation and sterile abscess formation in human tissues. The severity depends on the injection site: closed compartments (e.g., finger, vitreous cavity) carry disproportionately grave consequences. This mechanism explains the catastrophic intraocular inflammation in the present case. From a safety perspective, these findings underscore the need for prominent warning labels on oil-adjuvanted vaccines, comprehensive education on safe needle handling for farmers and veterinarians, and mandatory immediate medical consultation after any needlestick injury involving such products.

In addition, careful differential diagnosis is essential in this condition and requires distinction from several other ocular entities. The primary and most critical consideration is infectious endophthalmitis. Infectious endophthalmitis constitutes an ophthalmic emergency, typically caused by bacterial or fungal invasion of intraocular tissues with rapid proliferation (15). It is characterized as a purulent and diffuse inflammatory process, commonly presenting with corneal edema, hypopyon, and other severe anterior segment findings (16), and it usually progresses rapidly. In contrast, the present case represents an immune-mediated inflammatory response triggered by vaccine exposure. The patient showed poor response to repeated courses of antibiotic therapy during the early stage, whereas clinical improvement was observed after surgical intervention combined with removal of the intraocular liquid foreign material and corticosteroid-based anti-inflammatory treatment. These features support exclusion of infectious endophthalmitis. Autoimmune uveitis should also be considered in the differential diagnosis. Although its clinical manifestations may overlap with those observed in this case, the patient lacked systemic signs or symptoms associated with autoimmune disorders such as Behçet disease or ankylosing spondylitis (17). Moreover, a clear history of syringe penetration and intraocular liquid foreign material injection was present, allowing autoimmune uveitis to be reasonably excluded. Finally, other forms of intraocular foreign body reactions, including those caused by plant material, iron, copper, or stone fragments, are known to induce complications such as rhegmatogenous retinal detachment, vitreous hemorrhage, sympathetic ophthalmia, and infectious endophthalmitis (18, 19). Long-term retention of metallic foreign bodies may further result in siderosis or chalcosis, leading to progressive structural damage within the eye (20, 21). In contrast, the oil-based adjuvant and inactivated viral components contained in the porcine vaccine are capable of eliciting a robust immune response, accounting for the more intense and complex inflammatory manifestations observed in this patient.

Accidental intraocular injection of a porcine vaccine is exceedingly uncommon. In the present case, inadvertent intravitreal administration of the vaccine resulted in a prolonged and complex diagnostic and therapeutic course, contributing to improved understanding of the underlying pathogenic mechanisms and providing valuable clinical insights. The distinctive feature of porcine vaccines lies in their dual capacity to induce direct physical injury and chemical toxicity to ocular tissues while simultaneously provoking a strong immune response, thereby amplifying intraocular inflammation.

This case serves as a stark reminder of the devastating consequences that can result from accidental self-inoculation with oil-adjuvant veterinary vaccines. From the patient’s perspective, the sudden onset of severe pain and vision loss following injection, the uncertainty of visual recovery, the need for emergency surgery, and the prospect of long-term follow-up impose an immense physical and emotional burden. Therefore, comprehensive educational programs for agricultural workers and veterinarians on safe needle handling should be implemented, prominent warning labels should be affixed to vaccine packaging stating that immediate medical attention is required following accidental human inoculation, and workplace safety protocols should be strengthened to reduce the occurrence of such incidents.

## 4 Limitations of this case presentation

It is important to note the limitations of this study. First, as a single case report, the generalizability of the conclusions is limited, and it cannot be used to infer the incidence or absolute risk of such events. Second, given the suspicion of immune-mediated inflammation, cytokine profiling (e.g., IL-6, IL-10), cell count, or flow cytometry of the ocular fluids would have strengthened the diagnosis. Likewise, vitreous tap for Gram stain, culture, or PCR would have been ideal to definitively rule out infectious endophthalmitis. However, these tests were not performed due to the emergency vitrectomy setting, the patient’s limited financial resources, and the lack of laboratory availability at our institution. Furthermore, as the primary therapeutic goal was to preserve ocular function, we could not obtain histopathological specimens. Formal psychiatric evaluation was not performed, which is a limitation. The clinical history supports accidental injury, but future similar cases should include psychological assessment to exclude intentional causes.

## 5 Conclusion

Management of such atypical intraocular liquid foreign bodies accompanied by immune-mediated inflammation requires prompt pars plana vitrectomy to ensure thorough removal of the offending material, in combination with appropriate corticosteroid therapy to suppress inflammatory and immune reactions. In addition, sustained elevation of intraocular pressure associated with this condition warrants careful and long-term monitoring and management.

## Data Availability

The original contributions presented in this study are included in this article/supplementary material, further inquiries can be directed to the corresponding author.
